# A non-linear association between AST/ALT ratio and 28-day mortality in critically ill elderly: evidence from a multicenter study

**DOI:** 10.1038/s41598-025-11220-6

**Published:** 2025-07-16

**Authors:** Ling Wang, Ping Jin, Yanping Hui, Libo Li, Yixuan Wang, Xiang Wu, Yihua Bai, Lei Lu, Hongfei Qiao, Qiaojun Zhang

**Affiliations:** 1 Department of Rehabilitation Medicine, The Second Affiliated Hospital, Xi’an Jiaotong University, Xi’an, China; 2 Department of Cardiology, The Second Affiliated Hospital, Xi’an Jiaotong University, Xi’an, China

**Keywords:** AST/ALT ratio, Critically ill elderly patients, Mortality, WBC, Mediation effect, Medical research, Risk factors

## Abstract

With the global aging population, the proportion of elderly patients in ICU continues to rise. The ratio of aspartate aminotransferase to alanine aminotransferase (AST/ALT) has been linked to unfavorable outcomes in several diseases, but its value in critically ill elderly patients remains unclear. Based on the eICU Collaborative Research Database, this study included 22,361 critically ill elderly patients aged ≥ 65 years. The connection between the AST/ALT ratio and 28-day all-cause mortality was assessed using multivariate Cox regression analysis, and generalized additive models were employed to investigate non-linear relationships. A mediation analysis was undertaken to investigate the mediating impact of white blood cell count (WBC). Following a comprehensive adjustment for confounding factors, the AST/ALT ratio showed a non-linear association with 28-day mortality, with an inflection point of 2.161. When the ratio was < 2.161, Every additional unit was linked to a 43.9% greater risk of mortality (HR 1.439, *P* < 0.0001); when the ratio was > 2.161, this association was not statistically significant. According to Kaplan–Meier analysis, a significantly lower survival probability was observed in patients with higher AST/ALT ratios. Mediation analysis revealed that WBC partially mediated the link between AST/ALT ratio and mortality, accounting for 4.17% of the total effect. In critically ill elderly patients, the AST/ALT ratio is independently linked to 28-day all-cause mortality, demonstrating a non-linear relationship. WBC is an important mediator of this association.

**Supplementary Information:**

The online version contains supplementary material available at 10.1038/s41598-025-11220-6.

## Introduction

Healthcare systems face significant challenges due to the unique demographic transition towards an older global population, particularly in critical care settings. The World Health Organization predicts that the number of people aged 60 and over worldwide will rise significantly from 1 billion in 2019 to 2.1 billion by 2050^1^. This demographic transformation is markedly reflected in intensive care unit (ICU) admissions patterns, where elderly patients (≥ 65 years) currently constitute 35–40% of total admissions, with super-elderly individuals (> 80 years) representing 14.8% of ICU populations^2^. Contemporary epidemiological data demonstrate that the mean age of ICU patients has progressively increased to 67 years^3^. The aging ICU demographic presents distinct clinical challenges characterized by complex multimorbidity patterns, with approximately 75% of elderly patients exhibiting multiple chronic conditions, diminished physiological reserves, and enhanced susceptibility to adverse drug interactions^4^. Resource utilization analysis reveals that elderly ICU patients require significantly longer hospitalization periods (mean difference: 2–3 days) and incur 30–50% higher healthcare costs compared to younger cohorts^5^. The socioeconomic implications extend beyond clinical parameters, exerting substantial pressure on healthcare insurance systems, family economics, and social welfare infrastructure^6^.

Aspartate aminotransferase (AST) and alanine aminotransferase (ALT) are two essential aminotransferases with distinct tissue distributions and cellular localizations. AST can be found in the mitochondria and cytoplasm of different tissues such as the liver, heart, brain, skeletal muscle, and kidney, thereby reflecting mitochondrial dysfunction and oxidative stress. In contrast, ALT is chiefly present in the cytoplasm within liver tissue, thus serving as a liver-specific marker^7^. The AST/ALT ratio, referred to as the De-Ritis ratio, was initially described by Fernando De Ritis^8^. Increasing evidence demonstrates an elevated AST/ALT ratio is strongly linked to adverse outcomes across multiple pathological conditions. This ratio is related to both the severity of function and the mortality in heart failure within cardiovascular diseases^9,10^, as well as ICU mortality following cardiac arrest^11^. In infectious diseases, elevated ratios are linked to disease severity and mortality in COVID-19 patients^12^ and predict poor prognosis in severe sepsis^13^. Furthermore, among elderly populations, elevated AST/ALT ratios correlate with increased in-hospital mortality^14^ and accelerated cognitive decline^15^.

Specifically, when the AST/ALT ratio is below 1.80, higher ratios are significantly correlated with increased mortality; however, this association reaches a plateau when the ratio exceeds 1.80^14^. This pattern indicates that the connection between the AST/ALT ratio and mortality risk varies across different threshold ranges. Furthermore, the link between the AST/ALT ratio and the risk of mortality demonstrates population-dependent variations, as evidenced by numerous studies. Notably, research has identified that low ALT levels correlate with elevated mortality risk, particularly in elderly populations^16,17^. Furthermore, elevated AST/ALT ratios have shown a significant association with increased cardiovascular mortality^18^. While evidence exists linking AST/ALT ratios to mortality risk in critically ill elderly populations, the relationship is characterized by considerable complexity and ongoing scientific debate.

Importantly, there remains a significant research gap, since no research has specifically examined the link between the AST/ALT ratio and mortality from all causes over 28 days in critically ill elderly patients. This underscores the need for future research to validate and further elucidate the correlation between AST/ALT ratios and medical outcomes in this vulnerable patient population.

## Methods

### Data source

Data for this analysis were sourced from the eICU Collaborative Research Database version 2.0 (eICU-CRD v2.0), a comprehensive critical care database developed through collaboration between the Laboratory for Computational Physiology at Massachusetts Institute of Technology (MIT) and Philips Healthcare. This multicenter database contains high-granularity clinical data from over 200,000 ICU admissions across 335 intensive care units at 208 hospitals throughout the United States between 2014 and 2015^19^. The database encompasses detailed clinical information including vital signs, diagnostic codes (ICD-9), severity scores, laboratory findings, medications, and treatment protocols, making it a valuable resource for observational research. Using the Philips Healthcare eICU program, all data were collected and stored electronically without manual intervention.

The utilized database is released under the Health Insurance Portability and Accountability Act (HIPAA) Safe Harbor provision. Access to the database was provided following the completion of the Collaborative Institutional Training Initiative (CITI) program ‘Data or Specimens Only Research’ course and certification by the PhysioNet review committee. Ping Jin, the author, was given access and managed the data extraction process (certification number: 62661740). The security framework, certified by Privacert in Cambridge, MA, meets the safe harbor criteria for reidentification risk. Given that all research data underwent comprehensive anonymization, the Institutional Review Board (IRB) at the MIT evaluated and determined that this study qualified for exemption from further ethical review and waived the requirement for obtaining informed consent from participants or their legal representatives. All experimental protocols were approved by the MIT IRB. All procedures in this study were strictly conducted in accordance with the ethical guidelines of the Declaration of Helsinki and adhered to the STROBE (Strengthening the Reporting of Observational Studies in Epidemiology) reporting standards.

### Study population

**Fig. 1:**
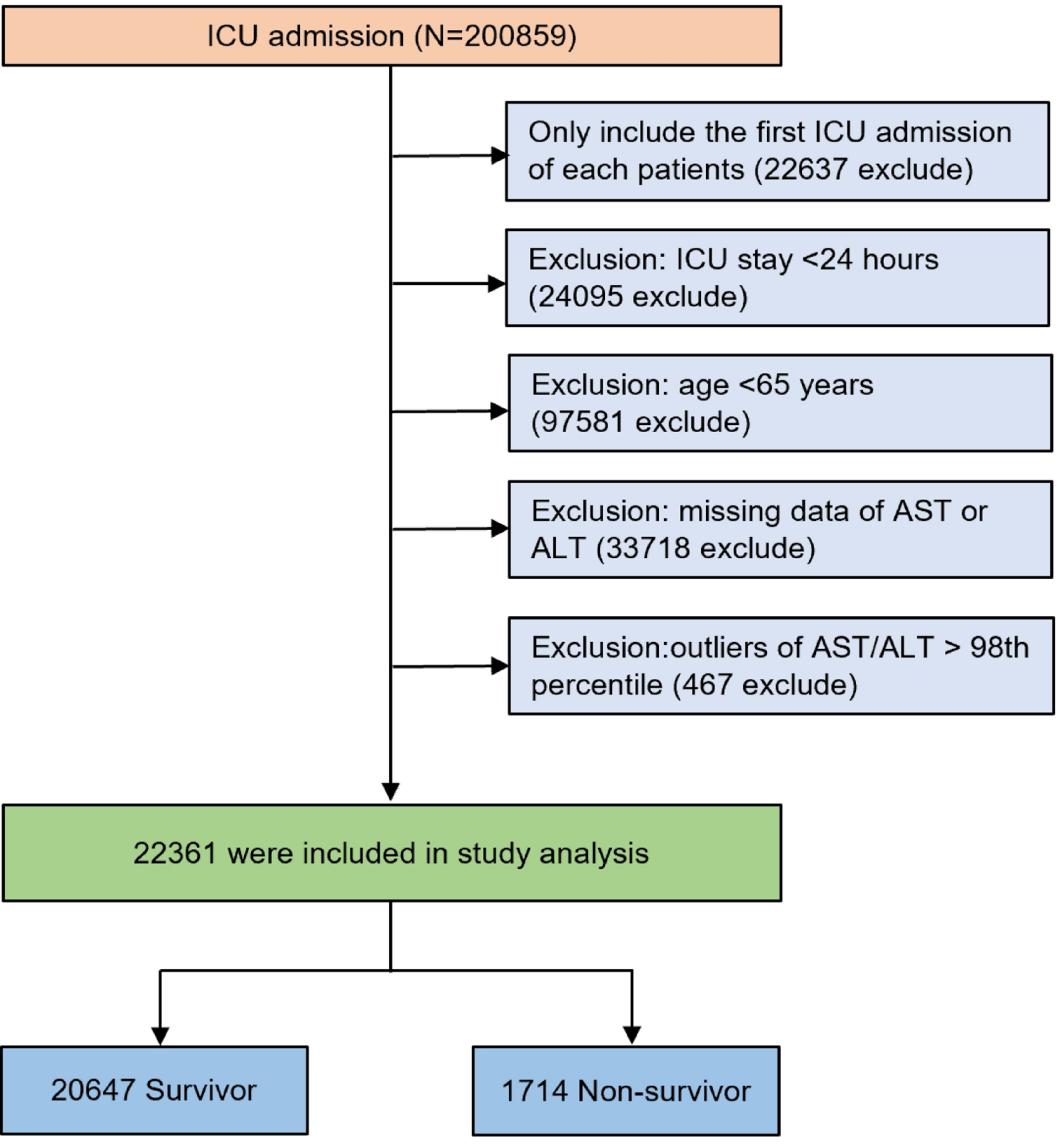
Flow chart of study population. *ICU* intensive care unit.

Every patient admitted to the ICU was evaluated for eligibility based on the following criteria. The exclusion process was conducted sequentially as follows: (1) non-first ICU admission; (2) ICU length of stay less than 24 h; (3) age below 65 years; (4) missing AST or ALT data; and (5) individuals with AST/ALT levels exceeding the 98th percentile. After applying these criteria, in the final analysis, 22,361 patients were included, with the study’s flowchart presented in Fig. 1.

### Data extraction

The following variables were extracted from the eICU-CRD database: (1) demographic characteristics: age, gender, ethnicity, and body mass index (BMI); (2) vital signs and laboratory parameters, such as temperature (°C), respiratory rate, heart rate, and mean arterial pressure (MAP), glucose, blood urea nitrogen (BUN), creatinine, albumin, platelets, red blood cell (RBC), WBC, hemoglobin, AST, and ALT; (3) comorbidities: pneumonia, acute myocardial infarction (AMI), diabetes (DM), cardiac arrhythmias and congestive heart failure (CHF); (4) severity scores: Glasgow Coma Scale (GCS) score, Acute Physiology Score III, and APACHE IV score. The calculation of the AST/ALT ratio involved dividing AST by ALT. All laboratory tests were performed using standard methods at each participating hospital’s clinical laboratory. The identification of comorbidities was based on ICD-9-CM codes extracted from the database. The severity scores were calculated using physiological parameters recorded during the first 24 h of ICU admission.

### Statistical analysis

Continuous variables were expressed with mean and standard deviation (SD), and categorical variables were represented by frequencies and percentages. A one-way ANOVA was used to evaluate differences in AST/ALT ratio tertiles for continuous variables, while a chi-squared test was applied for categorical variables. Multiple regression analyses were performed to evaluate the link between AST/ALT ratio and mortality. Three models were constructed with progressive adjustment for potential confounders identified through univariate analysis, literature review, and clinical relevance^10,14,20^. Model 1 presented unadjusted estimates. Model 2 was adjusted for demographic characteristics including age, gender, and ethnicity. Model 3 was further adjusted for clinical parameters including respiratory rate, heart rate, MAP, GCS score, AMI, pneumonia, and laboratory indices (creatinine, albumin, white blood cell count, and hemoglobin). Sensitivity analyses were conducted to determine the strength of the results. We managed missing data by using multiple imputation, which involved 5 replications and a chained equation method in the R MI procedure.

We employed a generalized additive model (GAM) to investigate potential non-linear relationships between AST/ALT ratio and mortality. When non-linear relationships were identified, we further applied two-piecewise linear regression models to determine the inflection points using likelihood ratio tests. Stratified analyses were conducted to assess the consistency of AST/ALT ratio effects across various subgroups, with interaction tests performed to evaluate effect modification. Results were visualized using forest plots. To evaluate survival outcomes, Kaplan–Meier survival curves were constructed, and differences in group survival distributions were analyzed using log-rank tests. Mediation analysis was conducted using the product of coefficients method to quantify the indirect effect of AST/ALT ratio on mortality mediated through WBC count, relative to the total effect. All statistical analyses were performed using R software (version 4.2.0) and Empower Stats (www.empowerstats.com, X&Y Solutions, Inc., Boston, MA, USA). A two-sided *P* value of less than 0.05 demonstrated statistical significance.

### Ethics approval and consent to participate

The data for this study was extracted from the eICU-CRD database in accordance with the data usage agreement (our record ID: 62661740) approved by the PhysioNet review committee. The database is provided under the HIPAA Safe Harbor provision. As this was a retrospective analysis utilizing an anonymized database intended for research purposes, ethical approval from the local ethics committee was not required. All experimental protocols were approved by the MIT IRB.

## Results

### Baseline characteristics of participants

In this retrospective analysis of 22,361 patients, we stratified the study population into tertiles based on AST/ALT ratio (0.075–1.043, 1.043–1.582, and 1.583–4.687) in Table 1, including 20,647 survivors and 1714 non-survivors, the comparison of these parameters between the two groups was shown in Table S1. Demographic characteristics across tertiles revealed a notable connection with increased AST/ALT ratios: advanced age (68.2 ± 14.7 vs. 62.4 ± 15.3 years, *P* < 0.001), male predominance (63.8% vs. 54.2%, *P* < 0.001), and lower BMI (23.8 ± 4.2 vs. 26.3 ± 4.8, *P* < 0.001). Regarding comorbidities, the prevalence of AMI increased significantly with higher AST/ALT ratios (28.7% vs. 18.4%, *P* < 0.001), while CHF (15.3% vs. 22.8%, *P* < 0.001) and DM (24.2% vs. 35.6%, *P* < 0.001) showed inverse associations. Disease severity indices demonstrated progressive elevation with increasing AST/ALT ratios, including Acute Physiology Score III (63.8 ± 25.4 vs. 48.2 ± 22.7, *P* < 0.001) and APACHE IV (82.6 ± 28.3 vs. 65.4 ± 25.8, *P* < 0.001), whereas GCS scores declined significantly (12.3 ± 3.8 vs. 14.2 ± 2.4, *P* < 0.001).

**Table 1:** The baseline characteristics of participants.

Variables	AST/ALT ratio			*P* value
	Tertile 1 (0.075–1.043) n = 7452	Tertile 2 (1.043–1.582) n = 7441	Tertile 3 (1.583–4.687) n = 7468	
Demographics				
Male (%)	3387 (45.46%)	3600 (48.39%)	3727 (49.93%)	< 0.001
Age (years)	75.62 ± 7.27	76.70 ± 7.53	76.76 ± 7.55	< 0.001
Ethnicity				< 0.001
Caucasian (%)	6155 (82.60%)	6000 (80.63%)	5975 (80.01%)	
Other (%)	1297 (17.41%)	1441 (19.37%)	1493 (19.99%)	
BMI (kg/m^2^)	28.61 ± 6.99	27.92 ± 6.81	27.47 ± 6.78	< 0.001
Comorbidities				
Pneumonia (%)	1217 (16.33%)	1150 (15.46%)	1151 (15.41%)	0.220
AMI (%)	252 (3.38%)	340 (4.60%)	718 (9.61%)	< 0.001
Arrhythmias (%)	1668 (22.38%)	1683 (22.62%)	1587 (21.25%)	0.099
CHF (%)	1009 (13.54%)	942 (12.66%)	858 (11.49%)	< 0.001
DM (%)	982 (13.18%)	862 (11.58%)	801 (10.73%)	< 0.001
Scoring systems				
Acute physiology score III	46.54 ± 19.78	49.48 ± 20.95	52.83 ± 22.18	< 0.001
GCS score	13.04 ± 3.18	12.63 ± 3.52	12.25 ± 3.77	< 0.001
Apache IV score	64.13 ± 20.45	67.60 ± 21.45	71.00 ± 22.75	< 0.001
Vital signs				
Temperature (°C)	36.42 ± 0.56	36.39 ± 0.63	36.36 ± 0.69	< 0.001
Respiratory rate (bpm)	26.22 ± 14.62	26.99 ± 14.83	27.25 ± 15.03	< 0.001
Heart rate (bpm)	98.87 ± 32.26	100.26 ± 32.01	102.44 ± 31.33	< 0.001
MAP (mmHg)	87.42 ± 43.68	84.64 ± 44.10	82.33 ± 44.64	< 0.001
Laboratory data				
Glucose (mg/dL)	145.99 ± 57.39	144.72 ± 56.96	141.46 ± 55.17	< 0.001
BUN (mg/dL)	29.02 ± 17.67	28.89 ± 17.39	30.13 ± 18.30	< 0.001
Creatinine (mg/dL)	1.40 ± 1.00	1.49 ± 1.04	1.63 ± 1.13	< 0.001
Albumin (g/dL)	2.86 ± 0.60	2.86 ± 0.64	2.80 ± 0.67	< 0.001
PLT (k/mcl)	201.83 ± 84.04	196.16 ± 82.60	189.14 ± 86.70	< 0.001
RBC (k/mcl)	3.69 ± 0.75	3.64 ± 0.72	3.57 ± 0.72	< 0.001
WBC (cells × 109/L)	11.49 ± 5.99	11.90 ± 6.20	12.75 ± 6.78	< 0.001
Hemoglobin (g/dL)	10.97 ± 2.26	10.82 ± 2.17	10.62 ± 2.17	< 0.001
AST (U/L)	44.89 ± 85.36	67.01 ± 156.24	102.80 ± 219.36	< 0.001
ALT (U/L)	60.97 ± 109.11	52.31 ± 120.63	45.37 ± 102.37	< 0.001

Among the 22,361 patients, the amount of missing values for the covariates were 5 (0.02%) for gender, 667 (2.98%) for BMI, 3285 (14.69%) for Acute Physiology Score III, 3290 (14.71%) for Apache IV score, 851 (3.81%) for GCS, 2289 (10.24%) for temperature, 640 (2.86%) for respiratory rate, 572 (2.56%) for MAP, 560 (2.50%) for heart rate, 516 (2.31%) for glucose, 875 (3.91%) for BUN, 606 (2.71%) for creatinine, 240 (1.07%) for albumin, 1056 (4.72%) for PLT, 832 (3.72%) for RBC, 988 (4.42%) for WBC, 632 (2.83%) for hemoglobin.

Elevated AST/ALT ratios demonstrated significant positive correlations with increased heart rate and respiratory rate, while MAP showed a decreasing trend (all *P* < 0.001). Laboratory analyses revealed that patients with higher AST/ALT ratios demonstrated significant hematological abnormalities, characterized by decreased hemoglobin levels, PLT counts, and elevated WBC counts, accompanied by impaired renal function as evidenced by elevated serum creatinine levels (all *P* < 0.001).

### Univariate logistic analysis

Univariate logistic regression analyses were conducted to examine the associations between clinical variables and 28-day all-cause mortality. As demonstrated in Table 2, higher levels of age, ethnicity, Acute Physiology Score III, Apache IV score, respiratory rate, heart rate, glucose, blood urea nitrogen, creatinine, AST, ALT, AST/ALT, and higher proportions of AMI and cardiac arrhythmias were significantly associated with increased 28-day all-cause mortality. Conversely, lower levels of BMI, GCS score, temperature, MAP, PLT, and albumin were significantly associated with increased 28-day all-cause mortality (all *P* < 0.05).

**Table 2:** The univariate logistic analysis between factors and 28-day mortality.

Exposure	Statistics	HR (95% CI)	*P* value
Demographics			
Male	10,714 (47.92%)	Ref	0.206
Female	11,642 (52.08%)	0.941 (0.855, 1.034)	
Age	76.36 ± 7.47	1.018 (1.012, 1.025)	< 0.001
Ethnicity			
Caucasian	18,130 (81.08%)	1.154 (1.02, 1.31)	0.025
Other	4231 (18.92%)	Ref	
BMI (kg/m^2^)	28.00 ± 6.87	0.986 (0.979, 0.993)	< 0.001
Comorbidities			
Pneumonia			
No	18,843 (84.27%)	Ref	0.053
Yes	3518 (15.73%)	1.116 (0.998, 1.247)	
AMI			
No	21,051 (94.14%)	Ref	< 0.001
Yes	1310 (5.86%)	1.341 (1.130, 1.592)	
Arrhythmias			
No	17,423 (77.92%)	Ref	< 0.001
Yes	4938 (22.08%)	1.290 (1.165, 1.429)	
CHF			
No	19,552 (87.44%)	Ref	0.760
Yes	2809 (12.56%)	1.021 (0.893, 1.168)	
DM			
No	19,716 (88.17%)	Ref	0.502
Yes	2645 (11.83%)	0.952 (0.826, 1.098)	
Scoring systems			
Acute Physiology Score III	49.62 ± 21.15	1.026 (1.024, 1.029)	< 0.001
GCS score	12.64 ± 3.52	0.896 (0.886, 0.906)	< 0.001
Apache IV score	67.59 ± 21.75	1.026 (1.024, 1.029)	< 0.001
Vital signs			
Temperature (°C)	36.39 ± 0.63	0.678 (0.632, 0.728)	< 0.001
Respiratory rate (bpm)	26.82 ± 14.83	1.012 (1.009, 1.016)	< 0.001
Heart rate (bpm)	100.53 ± 31.90	1.007 (1.005, 1.008)	< 0.001
MAP (mmHg)	84.79 ± 44.19	0.998 (0.997, 0.999)	0.003
Laboratory data			
Glucose (mg/dL)	144.06 ± 56.54	1.003 (1.002, 1.004)	< 0.001
BUN (mg/dL)	29.35 ± 17.80	1.012 (1.010, 1.014)	< 0.001
Creatinine (mg/dL)	1.51 ± 1.06	1.210 (1.168, 1.254)	< 0.001
Albumin (g/dL)	2.84 ± 0.64	0.684 (0.635, 0.736)	< 0.001
PLT (k/mcl)	195.69 ± 84.62	0.999 (0.999, 1.000)	0.003
RBC (k/mcl)	3.63 ± 0.73	1.005 (0.942, 1.073)	0.871
WBC (cells × 109/L)	12.05 ± 6.35	1.033 (1.027, 1.040)	< 0.001
Hemoglobin (g/dL)	10.80 ± 2.20	0.999 (0.978, 1.021)	0.918
AST (U/L)	71.59 ± 164.89	1.001 (1.001, 1.001)	< 0.001
ALT (U/L)	52.88 ± 111.13	1.002 (1.002, 1.002)	< 0.001
AST/ALT	1.45 ± 0.76	1.406 (1.335, 1.481)	< 0.001

*HR* hazard ratios, *CI* confidence interval.

No statistically significant associations were observed between 28-day all-cause mortality and other clinical variables, including gender, CHF, DM, pneumonia, RBC, and hemoglobin levels, among elderly critically ill patients following ICU admission.

### Multivariate Cox proportional hazards regression in different models

The relationship between the AST/ALT ratio and all-cause mortality within 28 days was evaluated using multivariate Cox proportional hazard regression models in Table 3. In the fully adjusted model (Model 3), each unit increase in AST/ALT ratio was associated with a 26.3% higher risk of 28-day mortality (HR 1.263, 95% CI 1.189–1.342, *P* < 0.0001). When categorized into tertiles, compared with the low AST/ALT ratio group (reference), patients in the middle tertile showed a 23.1% increased risk (HR 1.231, 95% CI 1.066–1.422, *P* = 0.0046), while those in the high tertile demonstrated a substantially elevated risk of 54.4% (HR 1.544, 95% CI 1.348–1.768, *P* < 0.0001). In every model, trend analysis consistently revealed a positive correlation between rising AST/ALT ratios and mortality risk, with a significant *P* for trend value (*P* < 0.0001).

**Table 3:** Association of AST/ALT ratio with 28-day all-cause mortality post-ICU admission in different models.

Exposure	Model 1		Model 2		Model 3	
	HR (95% CI)	*P*	HR (95% CI)	*P*	HR (95% CI)	*P*
AST/ALT	1.406 (1.335, 1.481)	< 0.0001	1.410 (1.338, 1.486)	< 0.0001	1.263 (1.189, 1.342)	< 0.0001
AST/ALT tertile						
Low	Ref		Ref		Ref	
Middle	1.337 (1.171, 1.528)	< 0.0001	1.320 (1.155, 1.508)	< 0.0001	1.231 (1.066, 1.422)	0.0046
High	1.962 (1.735, 2.218)	< 0.0001	1.942 (1.717, 2.197)	< 0.0001	1.544 (1.348, 1.768)	< 0.0001
*P* for trend	1.409 (1.327, 1.497)	< 0.0001	1.404 (1.321, 1.491)	< 0.0001	1.244 (1.164, 1.330)	< 0.0001

Model 1: We did not adjust for model variables. Model 2: include adjustments for age, gender, and ethnicity. Model 3: include adjustments for age, gender, ethnicity, respiratory rate, heart rate, MAP, AMI, pneumonia, GCS score, creatinine, albumin, WBC, hemoglobin.

Results from the sensitivity analyses corroborated the findings of our primary analysis, supporting the stability of our conclusions. Missing covariate data were handled using indicator variables. After accounting for the potential impact of missing data (Tables S2 and S3), the findings remained consistent. To further validate the robustness of our findings, we conducted additional statistical analyses after excluding patients with hepatic failure or cirrhosis (1042 patients in total), including smoothed curve fitting and multivariate regression analysis. The results of these additional analyses were highly consistent with our primary findings, indicating that the association between AST/ALT ratio and mortality in elderly ICU patients is robust and not significantly affected by underlying liver disease status (Table S4 and Fig. S1). These comprehensive analyses demonstrated the robustness of our primary results.

### Nonlinear relationship

**Fig. 2:**
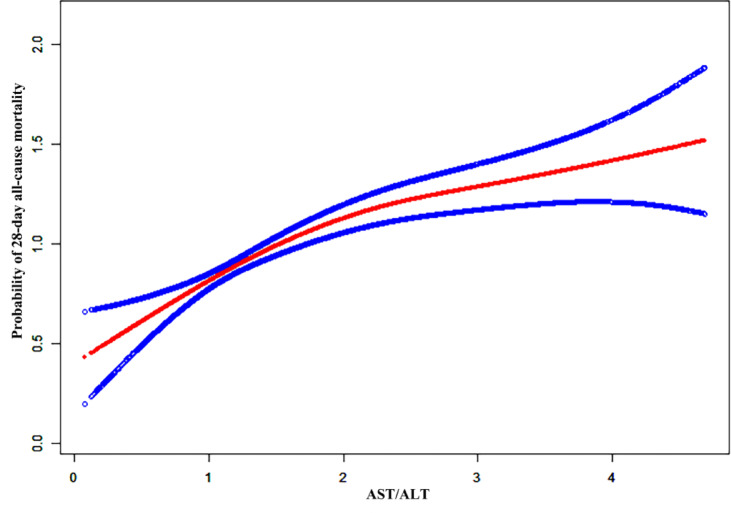
The generalized additive model revealed a nonlinear link between the AST/ALT ratio and 28-day all-cause mortality, with adjustments made for all variables in Models 3. Estimated values are depicted by the red lines, and their 95% confidence intervals are shown by the blue lines.

To examine the possible nonlinear link between the AST/ALT ratio and all-cause mortality within 28 days in elderly patients admitted to ICU, we employed smooth curve fitting analysis and GAM. The analysis, as depicted in Fig. 2, revealed a nonlinear association characterized by a clear threshold effect between AST/ALT ratio and 28-day mortality. As shown in Table 4, in the single-line effect model (Model I), each unit increase in AST/ALT ratio was connected to a 26.3% greater risk of dying within 28 days (HR 1.263, 95% CI 1.189–1.342, *P* < 0.0001), while each standard deviation increased corresponded to a 19.6% elevated risk (HR 1.196, 95% CI 1.141–1.252, *P* < 0.0001).

**Table 4:** Threshold effect analysis of the AST/ALT ratio and 28-day all-cause mortality.

Models	Per-unit increase		Per-SD increase	
	HR (95% CI)	*P* value	HR (95% CI)	*P* value
Model I				
One line effect	1.263 (1.189, 1.342)	< 0.0001	1.196 (1.141, 1.252)	< 0.0001
Model II				
Turning point (K)	2.161		0.925	
AST/ALT ratio < K	1.439 (1.283, 1.614)	< 0.0001	1.321 (1.210, 1.442)	< 0.0001
AST/ALT ratio > K	1.078 (0.941, 1.235)	0.2787	1.059 (0.954, 1.175)	0.2791
*P* value for LRT test*		0.0080		0.0080

Data were presented as HR (95% CI) *P* value. Model I: linear analysis. Model II: non-linear analysis. Adjusted for all variables in Models 3. LRT for logarithm likelihood ratio test. A *P value below 0.05 indicates a significant distinction between Model II and Model I.

More importantly, the threshold effect analysis (Model II) revealed significant nonlinear relationships with distinct turning points under different measurement scales. When analyzed by original units, a critical threshold was identified at AST/ALT ratio of 2.161. Below this threshold, each unit increment in the AST/ALT ratio was associated with a 43.9% increased risk of mortality (HR 1.439, 95% CI 1.283–1.614, *P* < 0.0001), whereas above this threshold, the association became statistically non-significant (HR 1.078, *P* = 0.2787). Similarly, when examined by standard deviation, a threshold effect was observed at 0.925, where each standard deviation increase below this point was associated with a 32.1% higher mortality risk (HR 1.321, 95% CI 1.210–1.442, *P* < 0.0001), while the association above this threshold was not statistically significant (HR 1.059, *P* = 0.2791). The likelihood ratio tests (both *P* = 0.0080) confirmed significant nonlinear relationships in both analyses.

### Subgroup analysis

**Fig. 3:**
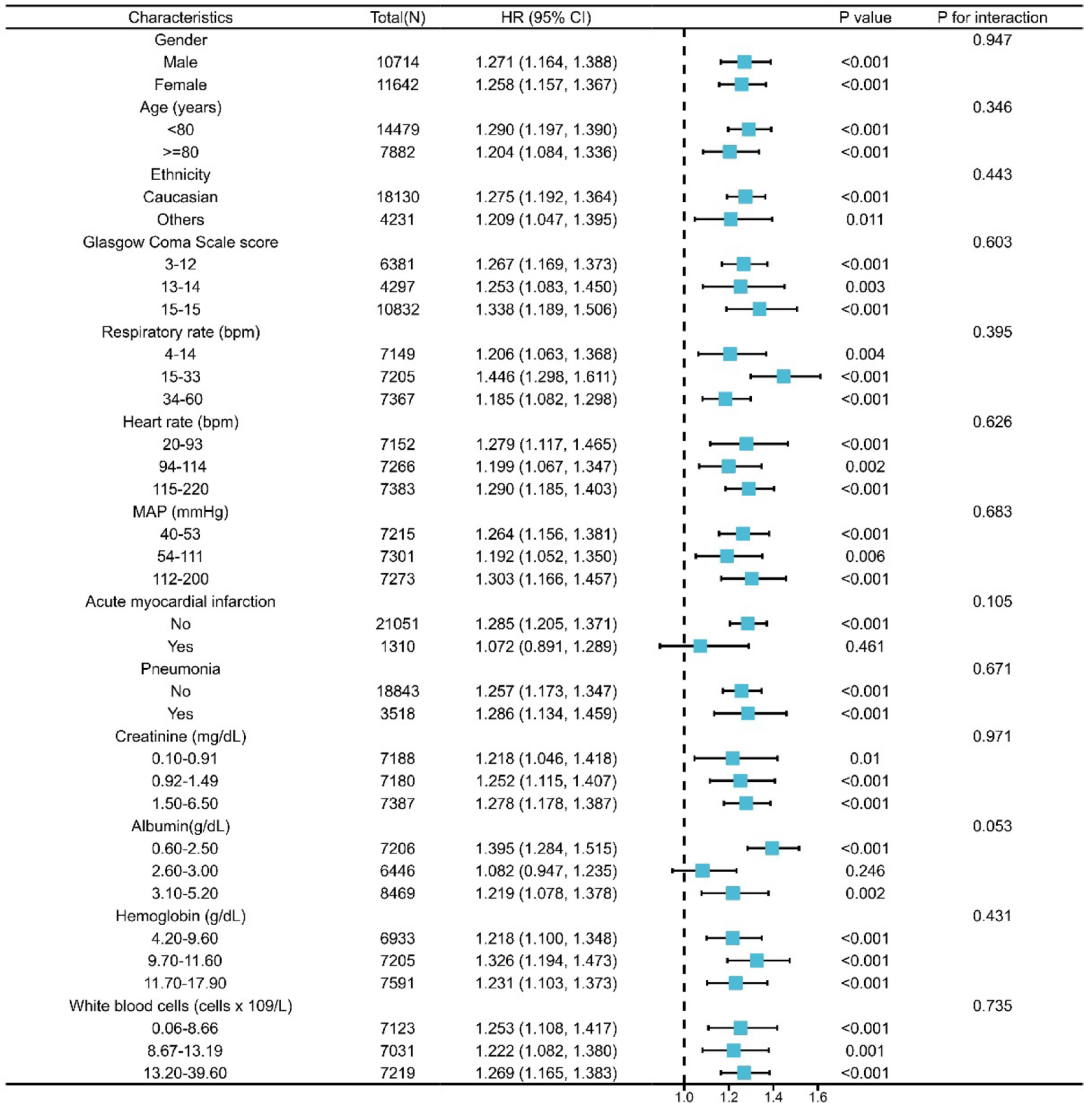
Association between AST/ALT and 28-day all-cause mortality in subgroups. Forest plot and adjusted HR (95% CI) for 28-day mortality. Apart from the stratification element, each stratification was modified to account for all variables in Models 3.

To assess whether the connection between AST/ALT and 28-day all-cause mortality might differ in various contexts, we executed a subgroup analysis focusing on age, gender, ethnicity, respiratory rate, heart rate, MAP, AMI, pneumonia, GCS score, creatinine, albumin, WBC, hemoglobin (Fig. 3). No significant effect modification was detected across the stratified analyses (*P* for interaction > 0.05).

### Survival curve analysis

**Fig. 4:**
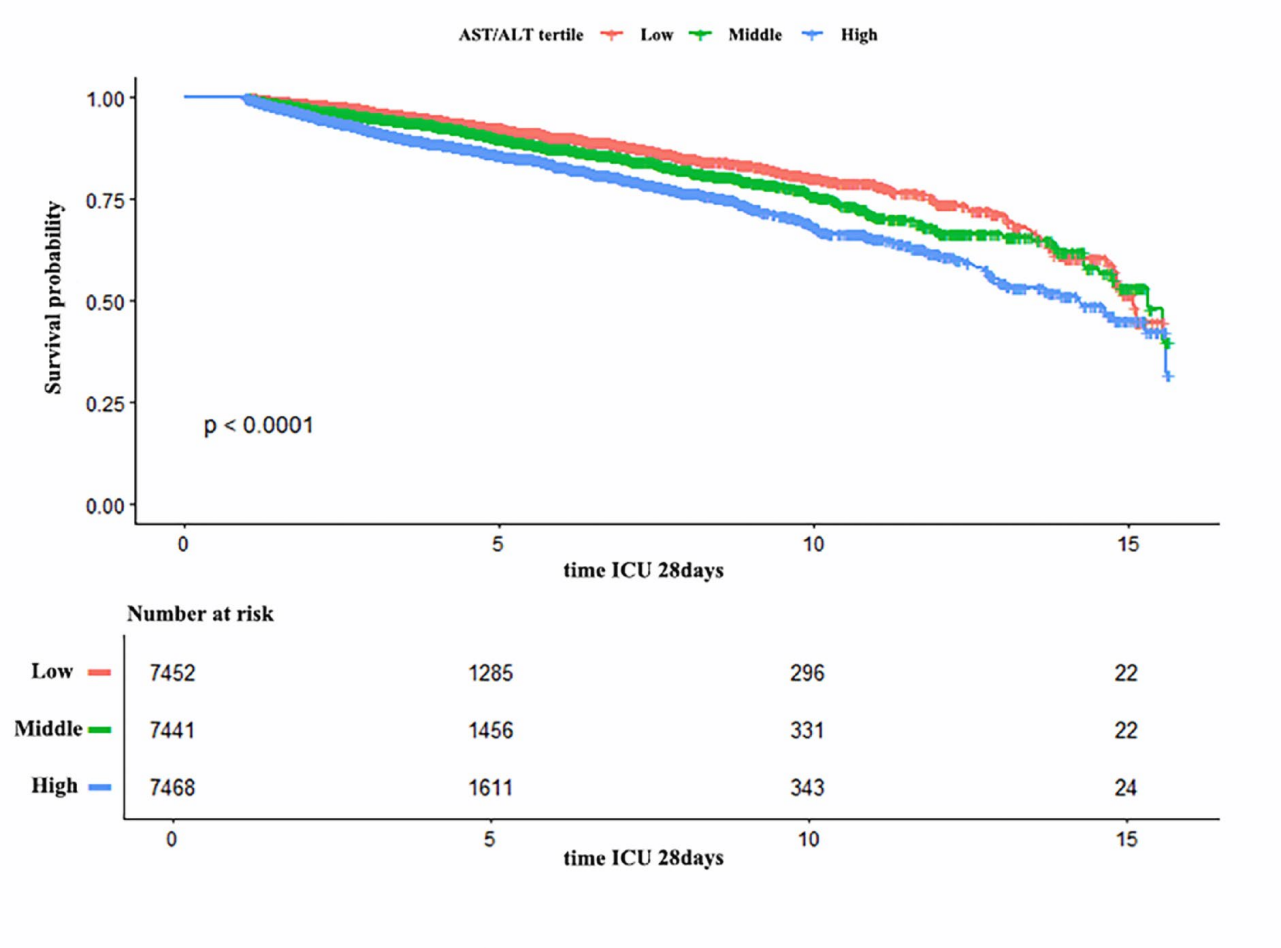
Kaplan–Meier survival analysis of elderly ICU patients with differing AST/ALT ratios. The ratio spans the following range: Low 0.075–1.043, Middle 1.043–1.582, High 1.583–4.687.

As depicted in Fig. 4, the Kaplan–Meier curves showed significantly different 28-day survival probabilities among the three AST/ALT ratio tertiles (log-rank test, *P* < 0.0001). Patients with AST/ALT ratios in the highest tertile (range 1.583–4.687) demonstrated markedly reduced survival probability compared to those in the lower tertiles.

### Mediation analysis

**Fig. 5:**
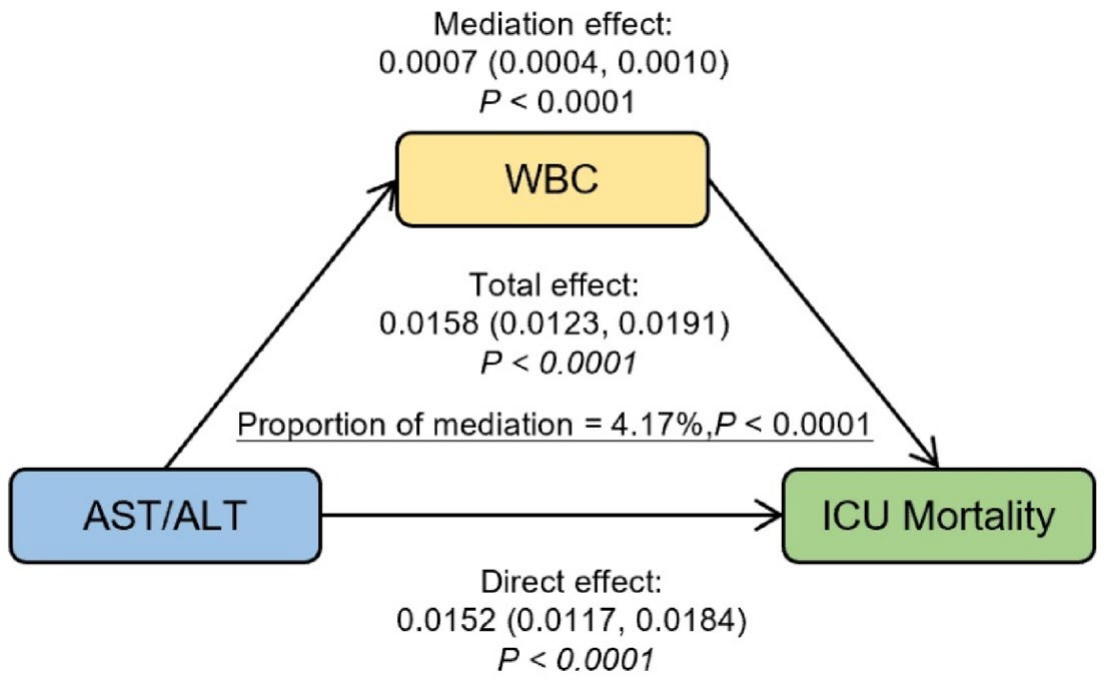
Effect of the WBC (mediator) on the relationship between AST/ALT (exposure) and ICU Mortality (outcome). WBC white blood cell.

Mediation analysis, as illustrated in Fig. 5, revealed that WBC partially mediated the relationship between AST/ALT ratio and ICU mortality (indirect effect: b = 0.0007, 95% CI 0.0004–0.0010). The analysis demonstrated that 4.17% of the total effect of AST/ALT ratio on ICU mortality was mediated through WBC levels. The above results were obtained after adjusting for all variables in Models 3.

## Discussion

In this large-scale multicenter study of 22,361 critically ill elderly patients, we demonstrated a significant and nonlinear link between the AST/ALT ratio and mortality from all causes over 28 days. Our findings revealed that elevated AST/ALT ratios were independently associated with increased mortality risk, with a threshold effect at 2.161. Below this threshold, each unit increase in AST/ALT ratio corresponded to a 43.9% higher mortality risk, while the association became non-significant above this threshold. This relationship remained robust after comprehensive adjustment for potential confounders and was consistent across various subgroup analyses. Notably, we identified WBC count as a partial mediator in this relationship, accounting for 4.17% of the total effect. Patients with higher AST/ALT levels demonstrated lower survival probabilities, as confirmed by Kaplan–Meier analysis.

This study demonstrated that elevated AST/ALT ratios were significantly associated with increased mortality in critically ill elderly patients. The pathophysiological mechanisms underlying this association are multifaceted and operate through several interconnected pathways: (1) From the perspective of liver dysfunction, elevated AST/ALT ratios serve as crucial indicators of hepatocellular injury^21^, and considering the inherently diminished hepatic compensatory capacity in elderly individuals^22^, this impairment significantly impacts multiple vital physiological processes. (2) The elevation of liver enzymes frequently coincides with systemic inflammatory states^23^, and given that elderly patients already exhibit compromised inflammatory regulation^24^, the release of inflammatory mediators may further exacerbate organ dysfunction. Moreover, liver dysfunction, as a critical component of multiple organ dysfunction syndrome^25^, not only affects coagulation through reduced hepatic synthetic function but also increases the burden on other organs due to metabolic disturbances. (3) Regarding pharmacotherapy, hepatic impairment significantly affects drug metabolism, potentially leading to drug accumulation or reduced therapeutic efficacy, thereby increasing treatment complexity and associated risks. As a crucial metabolic organ, liver dysfunction affects protein synthesis and energy metabolism, elevating the risk of malnutrition in elderly patients^26^. Additionally, the liver plays a vital role in immune regulation^27^, and its dysfunction increases infection susceptibility, particularly detrimental to elderly patients with already compromised immune functions. (4) Furthermore, liver dysfunction influences the metabolism of vasoactive substances, potentially exacerbating tissue hypoperfusion and affecting vital organ blood supply^28^. These mechanisms exhibit complex interactions, often creating vicious cycles that are particularly problematic for elderly patients with reduced physiological reserves and compromised compensatory capabilities, leading to cascade reactions and poor prognosis. Notably, the magnitude and manifestation of these associations may be modulated by various factors, including the type and severity of underlying diseases, comorbidities, nutritional status, and therapeutic regimens^29,30^. This underscores the necessity for individualized assessment and intervention strategies in clinical practice.

According to previous research, a particularly noteworthy investigation analyzed MIMIC-IV database focusing on critically ill elderly patients (aged ≥ 65 years) uncovered a complex, non-linear relationship between the AST/ALT ratio and in-hospital mortality (18.8%). The study revealed that when the ratio is below 1.80, each unit increase corresponds to a 39% higher risk of mortality. However, the relationship demonstrates a plateau effect when the ratio exceeds 1.80, suggesting a threshold effect in the association between the AST/ALT ratio and mortality risk in this population^14^. Our findings aligned with this study, showing that elevated AST/ALT levels were linked to increased mortality in critically ill elderly patients. In addition, our study demonstrates several advantages. While the prior study utilized the single-center MIMIC-IV database with 13,358 patients and an 18.8% in-hospital mortality rate, our study leveraged the multi-center eICU-CRD database encompassing 335 ICUs across 208 hospitals, with a substantially larger cohort (22,361 patients) and a lower mortality rate (7.7%). Most notably, our study introduced a novel mediation analysis, revealing that WBC counts partially mediated the relationship between the AST/ALT ratio and ICU mortality, accounting for 4.17% of the total effect. This innovative analytical approach not only expanded upon previous findings but also provided new mechanistic insights into how the AST/ALT ratio influences patient outcomes.

It is noteworthy that elderly ICU patients represent a highly heterogeneous population, and the clinical significance of the AST/ALT ratio may vary substantially among different disease subgroups. Recent studies have demonstrated that laboratory parameters may have different values across various critical illness phenotypes^31,32^. For instance, Palmowski et al.^33^ found that the AST/ALT ratio could be integrated with patient subphenotype analysis for mortality risk stratification in sepsis-associated acute liver injury patients. In the assessment of liver dysfunction, different definitions and clinical contexts may lead to variations in prognostic implications^34^. Particularly in patients with alcohol-related hepatitis, the severity of organ dysfunction at admission represents a primary risk factor for mortality^35^. Among patients with sepsis and septic shock, elevated AST/ALT ratio within the first 24 h of ICU admission has been independently associated with 28-day all-cause ICU mortality^36^. Yang et al.^37^ further emphasized the importance of considering subphenotype heterogeneity in critical care management by identifying clinical subphenotypes of sepsis following laparoscopic surgery^36^. These findings suggest that future research should explore the relationship between AST/ALT ratio and mortality in specific disease subgroups (such as hepatitis, septic shock, myocardial infarction, etc.) to more precisely evaluate its value and potential applications across different clinical contexts.

Although our observational study established a significant association between elevated AST/ALT ratio and increased mortality in elderly ICU patients, establishing causal relationships remains an important scientific objective. Traditional observational studies often struggle to demonstrate causality due to limitations such as unmeasured confounding factors and selection bias. In recent years, various advanced causal inference methodologies have emerged to bridge the gap between association and causation in observational research. Yang et al.^38^ provided an implementation method for the target trial emulation framework to evaluate fluid resuscitation strategies in patients with septic shock after laparoscopic surgery. Additionally, other causal analysis methods include instrumental variable analysis^39^, difference-in-differences methods^40^, propensity score matching^41^, Mendelian randomization^42^, and causal mediation analysis^43^. Each method offers distinct advantages: instrumental variable methods infer causal relationships using variables related to exposure but unrelated to outcomes; propensity scores simulate random allocation by balancing characteristics between treatment and control groups; and structural equation models provide frameworks for quantifying complex causal relationships^44^. In future research examining the relationship between AST/ALT ratio and mortality, these causal inference frameworks will help transform correlative findings into clinical insights with stronger causal foundations.

Our study demonstrates several significant clinical implications. First, we focused on a specific population of critically ill elderly patients with a large sample size (n = 22,361) from multiple centers (208 hospitals, 335 ICUs), ensuring robust representation. Second, we identified a novel non-linear relationship between AST/ALT ratio and 28-day all-cause mortality in elderly ICU patients, establishing a critical threshold of 2.161, below which each unit increase in AST/ALT ratio was associated with a 43.9% higher mortality risk. Third, these findings provide a valuable tool for early risk identification and disease progression monitoring, creating a window of opportunity for timely clinical interventions. Fourth, our mechanistic exploration revealed that WBC count partially mediates the relationship between AST/ALT ratio and mortality, offering new insights into the underlying pathophysiological mechanisms. Finally, these results can assist clinicians in risk stratification, facilitate personalized treatment planning, optimize resource allocation, and potentially improve patient outcomes. Collectively, these findings provide valuable guidance for critical care practice, particularly in the management of elderly ICU patients.

Our study has several limitations. First, as our study population predominantly consisted of Caucasian patients, caution should be exercised when generalizing these findings to other racial or ethnic groups. Second, our findings may not be fully representative of elderly patients who did not require ICU admission, and the mortality assessment was limited to 28 days post-admission. Third, while we adjusted for measurable confounding factors, the effect of unmeasured confounders in this observational study cannot be entirely ruled out. For example, therapeutic interventions such as mechanical ventilation, vasopressor use, and renal replacement therapy were not included in our analysis. Fourth, the lack of long-term follow-up data in our database precluded the assessment of the relationship between AST/ALT ratio and long-term mortality. Fifth, our study was based solely on a public database and lacks extensive validation. Sixth, our cross-sectional design, which did not analyze longitudinal changes in AST/ALT ratios, represents another limitation. Seventh, elderly ICU patients represent a highly heterogeneous population, and future research needs to explore the relationship between AST/ALT ratio and mortality in specific disease subgroups (such as hepatitis, septic shock, myocardial infarction, etc.). Furthermore, the exclusion of patients with missing AST/ALT values in our study may have introduced selection bias. This could affect the generalizability of our findings to the broader elderly ICU patient population. Finally, given the observational nature of our study, we could only establish associations rather than causal relationships between the variables of interest.

## Conclusion

This study is the first to demonstrate an independent association between elevated AST/ALT ratios and increased 28-day all-cause mortality in critically ill elderly patients using the eICU database. Higher AST/ALT ratios were consistently associated with lower survival probabilities, with a significant threshold effect observed. WBC was identified as an important modifier of this relationship. These findings highlight the need for close monitoring of AST/ALT ratios in elderly ICU patients. Additional future studies are necessary to verify these findings and investigate possible mechanisms and interventions to enhance outcomes.

## Electronic supplementary material

Below is the link to the electronic supplementary material.

Supplementary Material 1

## Data Availability

Data were fully available at https://eicu-crd.mit.edu/. Access to the database was provided following the completion of the CITI program ‘Data or Specimens Only Research’ course and certification by the PhysioNet review committee. Ping Jin, the author, was given access and managed the data extraction process (certification number: 62661740). The datasets used and/or analysed during the current study available from the corresponding author on reasonable request.
