# Supplementary material for: A non-linear association between AST/ALT ratio and 28-day mortality in critically ill elderly: evidence from a multicenter study
Source: Sci Rep. 2025 Jul 16;15:25831. doi: 10.1038/s41598-025-11220-6 (PMC12267599; doi:10.1038/s41598-025-11220-6)
Supplement: Supplementary file 1 — Supplementary Material 1 [file 41598_2025_11220_MOESM1_ESM.docx]

**Supplementary Information**

| **Table S1**. Baseline characteristics of participants grouped by survival status. | | | |
| --- | --- | --- | --- |
| **Variables** | **Survivor**  **n = 20647** | **Non-survivor**  **n = 1714** | ***P* value** |
| **Demographics** |  |  |  |
| Male (%) | 9894 (47.93%) | 820 (47.90%) | 0.981 |
| Age(years) | 76.32 ± 7.47 | 76.83 ± 7.41 | 0.005 |
| Ethnicity | | | 0.104 |
| Caucasian (%) | 16715 (80.96%) | 1415 (82.56%) |  |
| Other (%) | 3932 (19.04%) | 299 (17.45%) |  |
| BMI (kg/m^2^) | 28.03 ± 6.84 | 27.64 ± 7.22 | 0.002 |
| **Comorbidities** |  |  |  |
| Pneumonia (%) | 3099 (15.01%) | 419 (24.45%) | <0.001 |
| AMI (%) | 1167 (5.65%) | 143 (8.34%) | <0.001 |
| Arrhythmias (%) | 4400 (21.31%) | 538 (31.39%) | <0.001 |
| CHF (%) | 2558 (12.39%) | 251 (14.64%) | 0.007 |
| DM (%) | 2427 (11.76%) | 218 (12.72%) | 0.235 |
| **Scoring systems** |  |  |  |
| Acute Physiology Score III | 48.11 ± 20.23 | 69.50 ± 22.88 | <0.001 |
| GCS score | 12.87 ± 3.30 | 9.87 ± 4.70 | <0.001 |
| Apache IV score | 66.06 ± 20.88 | 87.75 ± 22.98 | <0.001 |
| **Vital signs** |  |  |  |
| Temperature (^o^C) | 36.40 ± 0.60 | 36.19 ± 0.86 | <0.001 |
| Respiratory rate (bpm) | 26.55 ± 14.80 | 30.09 ± 14.87 | <0.001 |
| Heart rate (bpm) | 99.65 ± 31.57 | 110.92 ± 33.93 | <0.001 |
| MAP (mmHg) | 85.13 ± 43.81 | 80.71 ± 48.29 | <0.001 |
| **Laboratory data** |  |  |  |
| Glucose (mg/dl) | 142.74 ± 55.07 | 160.27 ± 70.24 | <0.001 |
| BUN (mg/dl) | 28.83 ± 17.60 | 35.69 ± 19.00 | <0.001 |
| Creatinine (mg/dl) | 1.47 ± 1.04 | 1.89 ± 1.21 | <0.001 |
| Albumin (g/dl) | 2.86 ± 0.63 | 2.60 ± 0.69 | <0.001 |
| PLT (k/mcl) | 196.11 ± 83.68 | 190.70 ± 95.09 | 0.005 |
| RBC (k/mcl) | 3.63 ± 0.73 | 3.62 ± 0.76 | 0.165 |
| WBC (cells x 109/L) | 11.85 ± 6.16 | 14.39 ± 7.99 | <0.001 |
| Hemoglobin (g/dL) | 10.81 ± 2.20 | 10.74 ± 2.26 | 0.056 |
| AST(U/L) | 63.37 ± 143.04 | 170.63 ± 312.51 | <0.001 |
| ALT(U/L) | 48.56 ± 100.60 | 104.91 ± 190.52 | <0.001 |
| AST/ALT | 1.43 ± 0.75 | 1.73 ± 0.85 | <0.001 |

Data are expressed as the means ± SDs, medians (interquartile ranges), or numbers (percentages).

| **Table S2. Distributions of variables with missing data, comparing the observed complete case data set to results from pooling the datasets with imputed variables from multiple imputation.** | | | |
| --- | --- | --- | --- |
| Variables | Number (%)  with missing data | Complete case  Median (Min - Max) | Multiple imputation  Median (Min - Max) |
| Gender | 5(0.02%) | 1(0-1) | 1(0-1) |
| Respiratory rate (bpm) | 640 (2.86%) | 29.00 (4.00-60.00) | 29.00 (1.36-208.62) |
| Heart rate (bpm) | 560(2.50%) | 104 (20-220) | 104 (20-316.33) |
| MAP (mmHg) | 572 (2.56%) | 61 (40-200) | 61 (14.56-334.35) |
| Creatinine (mg/dl) | 606 (2.71%) | 1.16 (0.1-6.5) | 1.16 (0.1-10.95) |
| Albumin (g/dl) | 240 (1.07%) | 2.9 (0.6-5.2) | 2.9 (0.6-5.2) |
| WBC (cells x 109/L) | 988 (4.42%) | 10.6 (0.06-39.6) | 10.6 (0.06-87.99) |
| Hemoglobin (g/dL) | 632 (2.83%) | 10.7 (4.2-17.9) | 10.7 (2.6-18.85) |
| GCS score | 851 (3.81%) | 15 (3-15) | 14 (2.7-56.84) |

MAP mean arterial pressure, WBC white blood cell, GCS Glasgow Coma Scale.

| **Table S3.** **Multivariate Cox proportional hazard regression of AST/ALT with mortality with imputed variables from multiple imputation.** | | |
| --- | --- | --- |
| Exposures | HR (95%CI) | P value |
| AST/ALT | 1.309 (1.229-1.373) | <0.0001 |
| AST/ALT tertile |  |  |
| Low | Ref | Ref |
| Middle | 1.206 (1.055-1.378) | 0.0061 |
| High | 1.588 (1.402-1.800) | <0.0001 |

HR for hazard ratio, CI for confidence interval.

| **Table S4. Multivariate Cox proportional hazard regression analysis for association between AST/ALT ratio and 28-day all-cause mortality after excluding patients with hepatic failure or cirrhosis in different models.** | | | | | | |
| --- | --- | --- | --- | --- | --- | --- |
| **Exposure** | **Model 1** | | **Model 2** | | **Model 3** | |
|  | **HR (95% CI)** | ***P*** | **HR (95% CI)** | ***P*** | **HR (95% CI)** | ***P*** |
| AST/ALT | 1.392 (1.320, 1.468) | <0.0001 | 1.399 (1.325, 1.476) | <0.0001 | 1.248 (1.172, 1.328) | <0.0001 |
| AST/ALT tertile | | | | | | |
| Low | Ref | | Ref | | Ref | |
| Middle | 1.340 (1.171, 1.533) | <0.0001 | 1.334 (1.164, 1.527) | <0.0001 | 1.243 (1.074, 1.438) | 0.0036 |
| High | 1.948 (1.719, 2.206) | <0.0001 | 1.939 (1.710, 2.199) | <0.0001 | 1.535 (1.336, 1.763) | <0.0001 |
| *P* for  trend | 1.403 (1.320, 1.492) | <0.0001 | 1.401 (1.317, 1.490) | <0.0001 | 1.238 (1.157, 1.325) | <0.0001 |


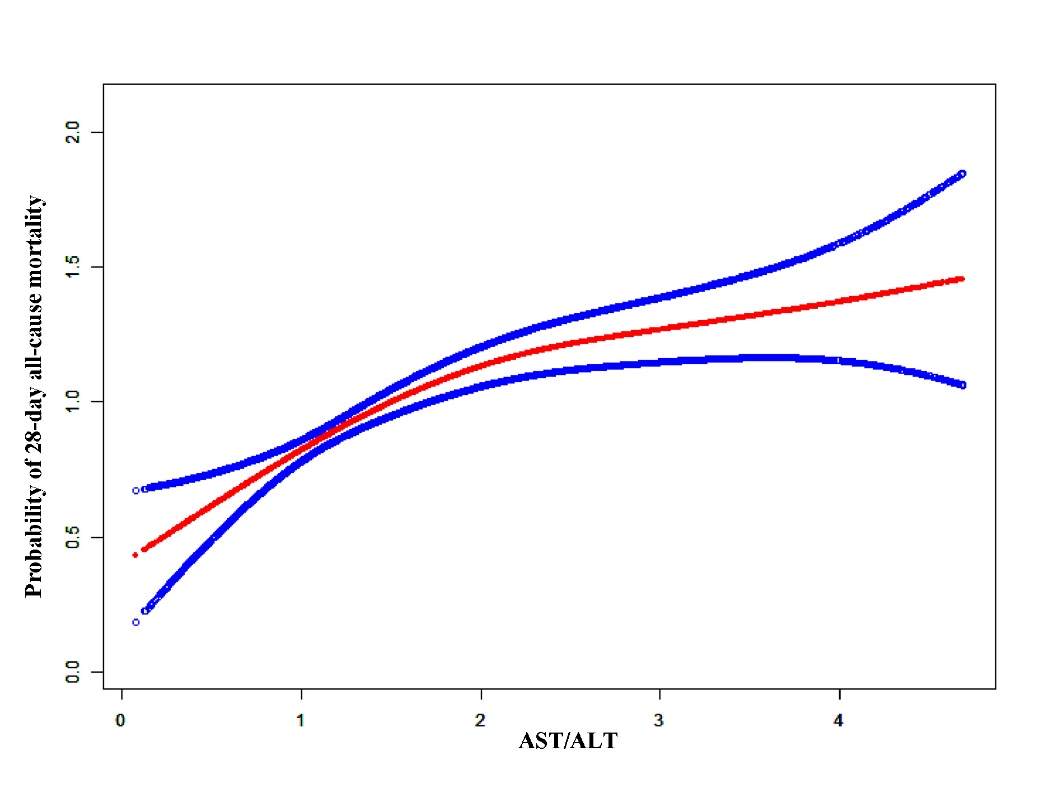
Data were presented as HR (95%CI) *P* value. Model 1: no adjustment for model variables. Model 2: adjust for gender, age and ethnicity. Model 3: adjust for gender, age, ethnicity, respiratory rate, heart rate, MAP, GCS score, AMI, pneumonia, creatinine, albumin, WBC, hemoglobin. MAP mean arterial pressure, GCS Glasgow Coma Scale, AMI acute myocardial infarction, WBC white blood cell. HR hazard ratios, CI confidence interval.

Figure s1. The generalized additive model revealed a nonlinear link between the AST/ALT ratio and 28-day all-cause mortality after excluding patients with hepatic failure or cirrhosis, with adjustments made for all variables in Models 3. Estimated values are depicted by the red lines, and their 95% confidence intervals are shown by the blue lines.
